# The Significance of Unifying Non-Integrated Information on Contaminated Land and Risks

**DOI:** 10.1007/s00267-025-02334-8

**Published:** 2025-12-27

**Authors:** Jacob Brotherton, Shoaib Hussain, Michael E. Deary, Charf Mahammedi, Cara D. Beal, Talib E. Butt

**Affiliations:** 1 Faculty of Engineering and Environment, Northumbria University, City Campus, Newcastle upon Tyne, Postcode: NE1 8ST UK; 2 School of Computing Engineering and Digital Technologies (SCEDT), Teesside University, Middlesbrough, TS1 3EB UK; 3 Griffith Institute for Human and Environmental Resilience and School of Environment and Science, Griffith University, Brisbane, QLD Australia

**Keywords:** Contaminated Land, Contaminated Sites, GIS Model, Special Sites, Information Management, Environmental communication

## Abstract

Like several countries with industrial history and heritage, the risks associated with contaminated land (CL) are a widespread challenge in the United Kingdom (UK). Contaminated land and their risks are managed by local authorities, although consultancy is often sought from non-governmental organisations. There is a legal requirement for the relevant local authority to hold data on the status of any contaminated land and associated risks within their geographical remit. However, minimal legislation exists regarding the standardisation of documentation, including records of contaminated site management and the associated administrative procedures. Another fundamental challenge is that the data concerning contaminated land is non-integrated. This is because hundreds of different local authorities up and down the country manage sites in a non-uniform manner. The same is the case in England and Wales, which is the geographical remit of this study. For instance, there are variations in format, type of data, presentation of data, procedures to access the data (e.g., by environmental consultants), quality of data, and even quantity of data. To overcome such issues presented by the lack of integration, there is a substantial need for unification and standardisation at all scales. This study not only presents an account of aforesaid issues and their adverse implications but also outlines innovative models that can enhance the integration, unification, simplification, and standardisation of data/information management from local authorities through to a national level. These conceptual models involve the application of information matrices and GIS. Such models, when fully developed in future, can enhance the environmental communication and coordination between the diverse stakeholders involved in each contaminated site scenario, particularly including environmental regulators e.g., the Environment Agency or Natural Resources Wales, environmental consultants, and local authorities; similarly other associated stakeholders such as developers, the construction industry and land reclamation specialists.

## Introduction

### Background

**Fig. 1:**
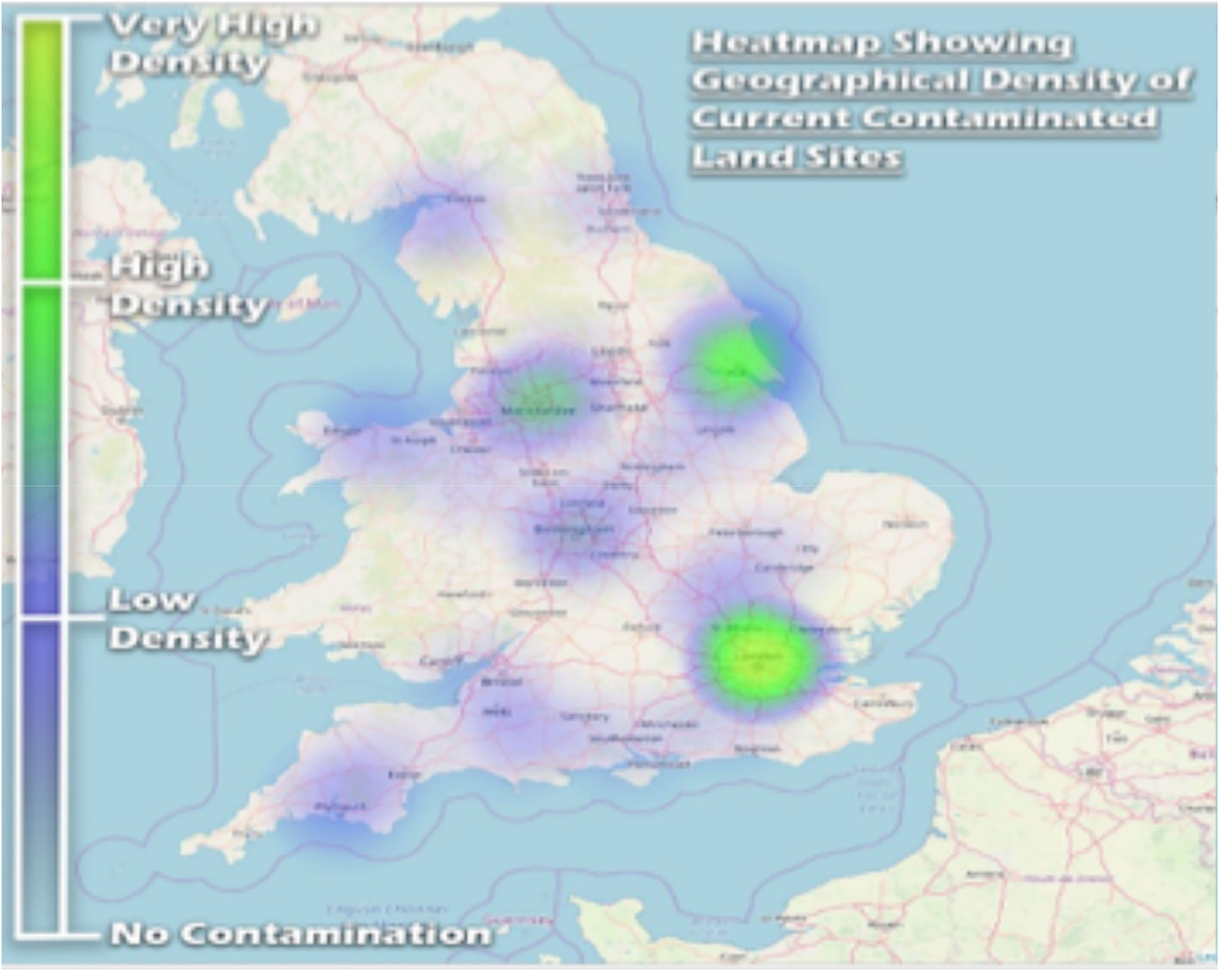
Heat map of the density of contaminated land sites

Industry in the UK began to decline in the mid-20^th^ century. In particular when, after the 1970s, the government introduced policy changes that deregulated markets, privatised industry, and shifted to commercialism (Foreman-Peck, 2006; Kitson & Michie, 2014). Base metal mining (e.g., tin, copper, and arsenic in regions like Cornwall and Devon, and lead, zinc, and copper in North Wales) reached its peak earlier than many industries, yet continues to pose contamination issues to this day. Industrial-scale coal mining developed later, played a significant role in the UK’s industrial landscape. Iron and steel working, which also contributed significantly to contamination, peaked in the mid-20th century (Douglas, Hodgson, & Lawson, 2002), as can be seen in the heat map of current contaminated sites (Fig. 1), which shows high concentrations in areas historically associated with these industries.

The Industrial Revolution transformed the composition of the UK economy from primarily agriculture and skill-based trades to large-scale industry, mechanised manufacturing, and the factory system. New machines, new power sources, and new manufacturing techniques made industries more productive and efficient. New industries also arose, including the automotive industry, steelwork, and mining (Britannica, 2019), all of which generated a great amount of chemical waste in all three states: solid, liquid, and gas. This waste was not managed in an environmentally friendly manner as profits were the main focus and the implications of waste were also misunderstood, which rendered the land contaminated (Das, 2020; Foster et al., 2021; Keeble, 2022; Salemdeeb, Al-Tabbaa, & Reynolds, 2016; Steinsson, 2020; Wardley-Kershaw & Schenk-Hoppé, 2022). There was large-scale de-industrialisation and the dereliction of industrial sites in the 1970s. As the UK economy reformed in the late 20^th^ century, contaminated ex-industrial sites began to be redeveloped, with the plan to reuse those sites after reclamation (Foreman-Peck, 2006; Kitson & Michie, 2014; Lawson, 2020; Stern, 2016; Stoklásek, 2022). The aim of redeveloping such sites was not just to secure the new economy, but also to seek environmental protection. This led to the implementation of the Environmental Protection Act (EPA) 1990, which targeted waste management and existing contaminated land (Catney, Henneberry, Meadowcroft, & Richard Eiser, 2006; Legislation, 2019; Luo, Catney, & Lerner, 2009; UK, 2011).

### Problem statement

The current governmental and publicly available data regarding contaminated land in England and Wales is spread across several individual databases, therefore requiring unification. There is no one set of documents relating to the extent of contaminated land at a national level (Catney et al., 2006; Ferguson, 1999; Nathanail, Bardos, & Nathanail, 2011; Rivett, Petts, Butler, & Martin, 2002). This poses the following main issues:

- An integrated national GIS (Geographic Information System) model which centralises local authority data on contaminated land does not exist.
- There is a substantial lack of integrated information to investigate issues surrounding contaminated land, from both human and environmental health perspectives.
- Both national and local governments are not able to effectively identify the remaining locations of contaminated land within England and Wales.

Even though the integration of contaminated land data is required at a national level, this should continue to be collected, organised, archived, and updated (as appropriate) by local authorities. However, there is a need for improvement in the management of such local data. All local authorities appear to hold a contaminated land public register in one form or another. Although this meets the legal requirements, it is often minimal in terms of accessibility and quality. In only around half of cases, is such data published online by local authorities. This demonstrates the variation, as well as inadequacy, in both the format and availability of information between local authorities. There is still a large amount of data that is inaccessible to the enquirer, be it governmental bodies, consultancies, or individuals. Due to the aforesaid issues, there is a lack of knowledge to inform decision making regarding contaminated land, be it land remediation, reclamation, or redevelopment.

From a national perspective, it would benefit a diverse range of stakeholders if data is combined and presented as a nationwide dataset to solve the issues posed by the non-integration mentioned above. National strategic policies regarding contaminated land lack effectiveness, due to the widespread and isolated nature of current local authority data. The absence of national statistics on contaminated land means there is next to no knowledge of the matter, which impedes the efficiency of national decision making.

### Aim and objectives

The main aim of this study is to design and develop innovative conceptual models that can demonstrate how to amalgamate, unify, and improve the presentation of the non-integrated data between local authorities on contaminated land (in England and Wales). The idea is to resolve the issues (identified in the problem statement above) arising from the isolated nature of these datasets. This would enhance the national governance of contaminated land, leading to more effective strategies and policies, with a higher degree of certainty.

This aim is managed via the following key objectives/research activities:

1. Establish the state of knowledge regarding the current management of contaminated land data for individual local authorities and examine the associated issues and challenges.
2. Analyse the current methods for determination, classification, and remediation (of contaminated land) that are being deployed by individual local authorities.
3. Identify and streamline the various terms regarding contaminated land.
4. Construct conceptual models in three forms:
  - A classification model for contaminated land
  - The amalgamation of local authority datasets into a holistic, interactive matrix.
  - The innovative GIS model to visualise and depict the holistic matrix.

### Scope and limitations

#### Management of data

This study focuses primarily on the presentation of contaminated land data. The initial focus of the study is on the history of, the current decision-making processes relevant to, and the legislation delineating contaminated land. This paper contains contaminated land data only from registers available online, as the purpose of this study is not to investigate beyond the public register but only to make the point that the unification is lacking among contaminated land data, information, administration, management and practices. The current management of contaminated land data is described (with insight into the issues surrounding this), the possibilities of local authority data availability/detail are listed, and shortcomings in current data availability are defined.

#### Geographical remit

Informational restrictions in Scotland and Northern Ireland confines the geographical remit of assessment and modelling to England and Wales.

#### Nuclear sites

As nuclear sites are managed under a different set of legislation and the responsibility lies with government bodies other than those who manage contaminated land for instance (Environment Agency), Nuclear Decommissioning Authority (NDA), Office for Nuclear Regulation (ONR), Defence Nuclear Safety Regulator (DNSR), and alike (Hill, 2009; ONR, 2018) –therefore, this paper focuses only on non-nuclear contaminated land (referred to simply as contaminated land).

## Methodology

The methodology employed in this study involved a comprehensive review of publicly available data from local authorities in England and Wales to assess the current state of contaminated land management. Data was collected in 2019 from local authority websites, focusing on contaminated land registers, remediation notices, and related documentation. This information was then organised into a holistic information matrix, capturing key details such as the number of contaminated sites, special sites, affected properties, grid references, contaminants, document links, and remediation status for each local authority. The matrix served as a centralised database for analysing and comparing contaminated land data across different regions.

To visualise the integrated data, a conceptual GIS model was developed. Grid references from the information matrix were used to create GIS layers representing current contaminated land sites, special sites, and sites with verified remediation. These layers were overlaid onto a UK map, providing a visual representation of the spatial distribution of contaminated land. Furthermore, a heat map was generated to show the spatial density of contaminated land sites, highlighting areas with a high density of contaminated sites. Statistical analyses were performed on the data extracted from the matrix and GIS model to derive insights into the extent, distribution, and characteristics of contaminated land in England and Wales.

## Existing practices and knowledge gaps

### The UK legislation

The presence of contaminants allows for the classification of an area as a contaminated site or as land affected by contamination, although these terms have no legal interpretation. A contaminated site becomes contaminated land if it meets the specific criteria detailed in Part 2 A of the EPA 1990, which contains all legislation regarding contaminated land. The legislation is categorised by letter; all contaminated land is under section 78 of the EPA. The sections in (Legislation, 2019) relevant to the scope of this paper are to be discussed below:

#### Section 78 A

This section defines contaminated land as any area with substances in, on or under the land where:

- Significant harm is being caused or there is a significant possibility of such harm being caused.
- Significant pollution of controlled waters is being caused or there is a significant possibility of such pollution being caused.

It is important to note the term “contaminated land” can describe an individual site, or more than one. The term is both singular and plural.

#### Section 78B

States that every local authority is legally required to inspect its respective area, to identify contaminated land sites, and that it must notify the appropriate agency and any appropriate persons involved.

Local authorities are primary investigators for contaminated land. However, the referral of information is required to be made to the above-mentioned appropriate agency. In England, this would be the Environment Agency (EA), and in Wales, Natural Resources Wales (NRW). These regulatory environmental bodies provide management and consultancy services to ensure local authorities adhere to guidelines.

#### Section 78 C

An area of contaminated land can be declared as a “special site” (SS) if deemed to be a serious risk. This section defines special sites as simply causing or having the potential to cause serious harm or pollution. The UK Government (Government, 2014a) expands on this by stating such sites include contaminated land which:

- Seriously affects drinking waters, surface waters or important groundwater sources.
- Has been, or is being, used for certain industrial activities, such as oil refining or making explosives.
- Is being or has been regulated using a permit issued under the integrated pollution control or pollution prevention and control regimes.
- Is contaminated by waste acid tars.
- Is owned or occupied by the Ministry of Defence.
- Is contaminated by radioactivity.
- Is a nuclear site.

The relevant local authority identifies sites that could meet one or more of the SS criteria and refers to the appropriate agency, who will advise on the designation as a special site.

#### Section 78E

By section 78E of 2 A, when contaminated land is designated, the local authority must produce a remediation notice. This is a document detailing what must be done to remediate the site. The document should reference the estimated cost of said remediation, the proportion of costs between stakeholders, the severity of potential harm, and the contaminants in question. Such documents are only prepared once land has been formally determined as contaminated under Part 2 A.

#### Section 78QA

Land that is no longer contaminated will have a non-contamination notice produced, which will be held on record, including details of the land and the date of designation as non-contaminated.

#### Section 78 R (Registers)

This section states that every local authority must hold a public register which contains many documents, including remediation notices, statements, declarations, and special site designations. It is also stated that there is a duty to maintain the registers for public inspection, free of charge. However, the registers are allowed to be kept in any form. It is important to note that “*the register is a permanent record of all regulatory action (e.g., remediation notices and statements) undertaken to ensure the remediation of any site which has been formally determined as contaminated land under Part 2* *A. Any land satisfactorily remediated prior to a remediation notice being served will not appear on the register. The register will also record the ‘Designation’ of Special Sites.”* (Council, 2019). This means many contaminated sites are not entered onto the register, as they may not meet contaminated land criteria, or have already been remediated through other methods before determined under Part 2 A.

There are three main ways in which land affected by contamination can be remediated: under Part 2 A of the EPA, through the planning system, or voluntarily – described below:

#### Under Part 2 A

By remediating under Part 2 A, the land must be legally determined as contaminated, the relevant documents will be produced and held on the public register.

#### Through the planning system

By remediating through the planning system, private bodies will decontaminate the site as part of a development application proposal, documents relating to such remedial efforts are not required to be kept on the register as they are not under Part 2 A.

#### Voluntarily

Voluntary remediation is where individuals or groups will remediate contamination within their land. Again, documents relating to this type of remediation do not need to be held on the public register.

Remediation may also be required upon the surrender of an Environmental Permit, as permit operators have a duty to prevent environmental damage. Therefore, the public registers may only have a record of contaminated land determined under Part 2 A. From the perspective of archiving and presenting contaminated land data, the aforesaid legislation does not appear sufficient in regulating comprehensive documentation for such data. This is a challenge which needs to be addressed. According to Natural Resources Wales (Wales, 2016), in Wales, 209 sites remediated between 2001 and 2013 were done so under Part 2 A, with over 5500 remediated through the planning system – where records may not be held.

### Governance of contaminated land

Contaminated land is formally identified by the local authority (council or borough) responsible for the geographical area in question. Councils and boroughs are simply geo-political zones of England and Wales that make up the full land area. Preliminary assessments are used to determine sites of potential land contamination. At a basic level, historical land-use maps are utilised by investigators to identify where contaminants may be present. These sites were, and are, predominantly industrial or military, with common sites listed by the government as factories, mines, steel mills, refineries and landfills. It is also likely that previous and current military zones are contaminated due to their high waste output of volatile fuels and other hazardous chemicals (Government, 2014a).

Land can be investigated by the local authority or appropriate agency for many reasons, for instance, investigations are commonly performed when uninvestigated land is sold; transferred or proposed for development when a general inspection is proposed; when an application for environmental or other permit is made; or when land is suggested to be polluted. Contamination must be dealt with before planning permission is given on that site (Government, 2014a). During the risk assessment and remediation process of CL there are four main steps (GeoSmart, 2018a):

- Desk study and site survey (initial identification of potentially contaminated sites).
- Determination (detailed site investigation and risk assessment, formal classification as CL or not).
- Remediation (planning and implementation of a remedial strategy).
- Verification (final risk assessment and site survey to determine the success of remediation).

#### Desk study and site survey

In connection to desk studies, historical land-use maps are utilised in contaminated land assessments to reconstruct past activities at a site, helping determine potential contamination sources and pathways. This is crucial because concerns can be raised about previous industrial or waste disposal sites, as contaminants are most likely present in the soils of such areas. By understanding previous land uses (e.g., industrial sites, landfills, gasworks), investigators can better target investigations and sampling, focusing on areas with a higher probability of contamination. This historical context is essential for understanding the nature, extent, and sources of contamination, and for assessing potential risks to human health and the environment, informing risk assessments, remediation strategies, and regulatory compliance. Site surveys, such as walkovers can identify specific places on site where contaminants may be present, with physical evidence of the previous land-use. Desk studies are also used to identify the geography and geology of the area, to help identify risk potential.

#### Determination

An on-site investigation is launched where data is collected for analysis. This data can be used as a reference for contaminant levels in soil layers and surface soil, groundwater and aquifers, and gas levels. After analysis, the local authority will determine whether the land in question is contaminated or not. If the land is found to be contaminated it can be formally determined, or if there is suspicion(s) of meeting special site criteria, the investigation may continue.

#### Remediation

The risks identified in the determination stage are analysed further, the local authority plans a remediation technique(s) to be used to remove one or more of the risk constituents (source, pathway, and receptor).

#### Verification

This is used to demonstrate the success of remediation, an investigation (like in the determination stage.) is undertaken, and the data created is used to validate that the site is now safe for use.

### Contaminated sites and the public register

There are several reasons for contaminated sites (which may or may not be contaminated land) to be absent from the public register, such as the remediation types mentioned above. However, it is possible that designated contaminated land is also unavailable on the public register, the plausible explanations for this are discussed below.

#### Contaminated land that has not yet been formally identified

Contaminated land that remains unidentified this late in the progression of the Part 2 A legislation will nearly always be insignificant. Local authorities have actively searched for contaminated land sites since the introduction of Part 2 A in 1990, the criteria surrounding these searches involves looking for areas of previous industry and where risk could be significant currently (such as property sites or water systems). It is within the remit of a local authority’s duty to investigate sites they identify with potential risk. The likelihood, almost 30 years after investigations began, of a non-examined site being contaminated, is very low, as it will have remained uninvestigated due to a perceived minimal risk. The other case is that the local authority has had no indication that a site is contaminated. It is of very low probability that a site of this nature could cause harm as it will be missing the contaminant linkages that amplify risk. If it did have the contaminant link, it would have already been identified by the local authority. Therefore, contaminated land that has not been formally identified is highly likely to be insignificant, either because of its low contaminant concentrations or its location being isolated from pathways and receptors.

#### Contaminated land that is still being processed

As entries on the register are only made when a remedial strategy is formalised, the local authority may have identified a site of significant risk and are yet to finish preparing their remediation plan. This means a site of significant risk exists but is unknown to the public. While this case sounds more significant, there are likely to be few such sites across the UK, and it is also a relatively short-term issue: the number of new sites being remediated at this stage of Part 2 A is low. For a lot of local authority data, their last remediation was over ten years ago. This shows that the number of contaminated land sites per council/borough is rather low in general, as it takes a significant risk to require remediation. Therefore, contaminated land that is still being processed can be thought of as significant in the relevant local authority, but on a national level, it makes very little difference as there will only be a handful of examples.

#### Sites redacted due to sensitive information

There will be few examples of this, and so it may have a little significance though the prospect of redacted sites being dangerous to the public, could be contentious. An example would be a site being used for confidential military options (but in this case it is unlikely to reach many receptors). In summary, UK councils must balance transparency and data protection when dealing with contaminated land information. They are subject to the Freedom of Information Act and Environmental Information Regulations, which allow public access but also have exemptions. Redacted data may include sensitive details about contamination locations or remediation strategies (Chen & Kirkham, 2024; Hagen). Individual council policies influence the level of data availability. Generally, summary information on contaminated sites is accessible, while specific details may be restricted in scenarios such as the site investigation being still underway (Latawiec, Simmons, & Reid, 2010). Therefore, the information regarding certain contaminated sites may be redacted due to their sensitive contents which could be significant but will be rare nationally.

### Identification and documentation of contaminated land

In this paper, only government and local authority sources are considered for the collection of data - being up to date and accurate. As mentioned above, there are often cases where contaminated sites are not entered onto the register. It is also found that, while local authorities claim to hold an up-to-date register, it is commonly not published online as it is kept at the local authority offices for viewing. In the case of special sites, a pre-existing government document is available with relevant information 2019 (Department for Environment, 2012). All gathered data is presented in the matrix model. Firstly, it is found that there is no single publicly available database for the location and nature of contaminated land at a national level. Some local authorities hold shapefiles (a visual database) of where sites are, they are GIS map layers describing site boundaries, like those published by Sheffield City Council (S. C. Council, 2020). While this shapefile data is available on the government database, there is a very small number of local authorities which have published them. Only a few of these local authorities have comprehensive data and even then, these shapefiles do not contain information on the type of contamination, only the location of sites. They are difficult to interpret and serve more as a visual aid. In other categories of environmental documentation, there are already national models similar to those suggested for contaminated land in this paper, such as historic landfill records (Department for Environment, 2019). The existence of national models highlights that there is already an established benefit to integrated data, yet this does not exist for contaminated land. So, in order to collect and compile contaminated land data, there must be an individual search across all English and Welsh council/borough databases of their contaminated land registers (if available).

To help give a better understanding of the collected data, the cases of data availability met during research are to be explored next:

#### No data available

In this case, there is no online record of the public register for the local authority in question. So, under these circumstances, the number of contaminated land sites is entered as “no data” in the information matrix.

#### Verified no contaminated sites

Almost as many local authorities as ones with no data available listed that they had no contaminated sites. This is almost always done in the form “while (council/borough name) has a public register, there are currently no entries”. Some included a copy of their empty public register to show this, others just stated that there is no contaminated land within their council/borough. In this case, the number of contaminated land sites is entered as “0” on the spreadsheet, meaning the land investigated has been verified as non-contaminated within that local authority.

#### Data available

In this case, the local authority has published its register data online, meaning the details can be accessed and extracted. There are two ways in which the local authorities showed data:

1. Some local authorities attached their public register, where site locations and the number of sites can be found. In these cases, there are often remediation and determination statements published alongside; though this is not always the case. Where remediation and determinations are attached, the contaminants and status of remediation could be obtained. Where they are not attached, the contaminants are listed as “no data” on the spreadsheet.
2. The second case being local authorities not uploading their public register but instead creating a summary of contaminated sites (the full register is only viewable in person, but the summary provides enough information to satisfy the criteria assessed in this paper). This is often in spreadsheet format, with information from a selection of site location, contaminants and remediation status available; the information varied per local authority and has been entered into the spreadsheet in conjunction with what is available.

## Development of innovative models

### Classification model

There is a need for the classification of land affected by contamination. Contaminated land can be categorised into one of three types listed below along with the corresponding colour coding:

- Current contaminated land sites (Orange colour)
- Current special sites (Red colour)
- Sites with verified remediation (Green Colour)

**Fig. 2:**
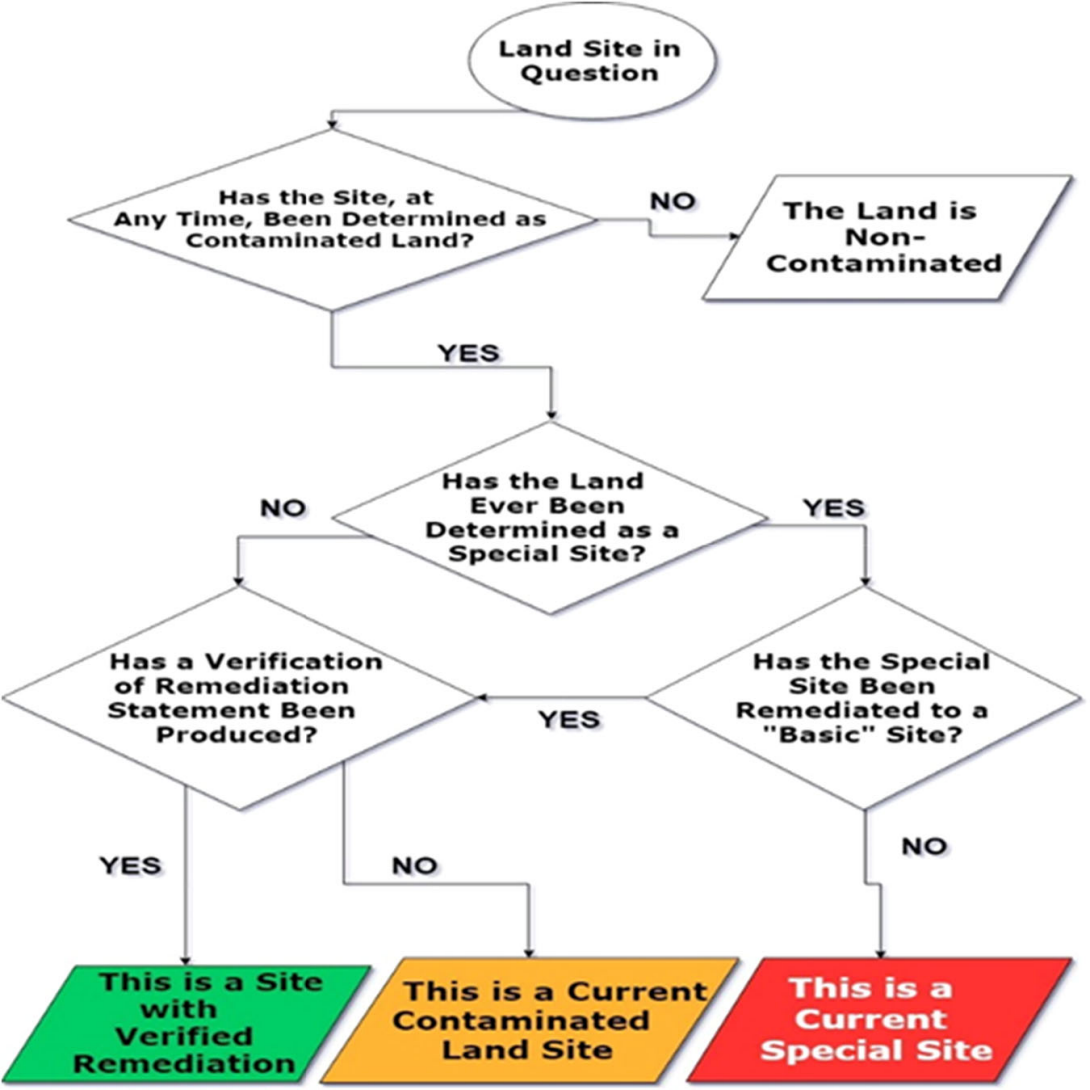
Flow chart model of contaminated land classification

To further clarify the decisions taken during classification, a flow diagram is shown below in Fig. 2.

This model outlines the pathway from basic identification to the classification of contaminated land into the three types. The types are mutually exclusive, meaning no individual site can fall into multiple types. Regardless of a given contaminated land site was special or not, the model is focusing only on the current state of site. The model acknowledges the history of the site while focusing on the current status. If the site was contaminated and now has been remediated (and verified) then the colour code is green. A somewhat fair assumption made in the classification is that where there is no verified remediation, the site is still to be deemed as contaminated, which means it be either orange (i.e., currently contaminated land site) or red (i.e., special contaminated site). This assumption is fair as if a site had been remediated, the local authority would release confirmation. If the site is contaminated and not special it is not necessary to refer to it as non-special contaminated land, though this can be done for clarification.

However, the difference between the two is explained as follows; contaminated land is categorised into ‘special sites’ and ‘non-special sites’ based on the severity and nature of the contamination, as well as who is best suited to regulate them (Hohner et al., 2020). Special sites involve contamination that can have serious consequences for human health and/or environmental health. In this context, a few typical examples of sensitive of environmental receptors are drinking water, surface water, or important groundwater sources, or involves certain industrial activities like oil refining or explosives manufacturing (Fent, 2004). These sites are regulated by environmental agencies, namely the Environment Agency in England, Natural Resources Wales, or the Scottish Environment Protection Agency (SEPA), rather than local authorities (Hill, 2009). On the other hand, non-special sites have typically less severe contamination issues, while are managed and regulated by the local authority in whose area the land is situated (Higgins, 2016). Furthermore, in the context of administration and management, the differentiation lies in the fact that sites with the most complex or hazardous contamination are handled by specialised agencies with the appropriate expertise and resources.(Swartjes, 2010).

### Holistic information matrix model

The collection of data from hundreds of local authority websites has allowed for the development of a data-rich interactive matrix. The matrix acts as a holistic database for all sites of registered contaminated land in England and Wales. Each row is a local authority within England and Wales, and the columns are informational sections – the headings of which are as follows:

- *The number of “basic” contaminated land sites*: the term “basic” is used to describe a contaminated land site determined under Part 2 A which is not a special site, hence only being a basic/normal site. This includes all determined sites of contamination, past or present, so some may no longer be classed as contaminated.
- *The number of special sites:* this is the number of areas currently identified as a special site, it is not inclusive of previous special sites as they are either removed from the register or are now basic contaminated sites. Where there is data, the value has been hyperlinked to the source of that information.
- *Total contaminated land sites:* this is the sum of the number of “basic” and special sites.
- *The number of properties affected:* this is the verified number of properties that have been affected by contamination, usually listed in the remediation strategy, this includes past and present properties within a contaminated land site. Property in this case as defined as individual residences or farmland, where there are no residences, the properties affected has been listed as 1; these are nearly always industrial sites.
- *Grid References:* this is a list of the national grid references (NGRs) for all the registered sites; there is often multiple per local authority.
- *Contaminants:* this is a list of the substances found on the site that are exceeding Soil Guideline Values (SGVs) or have been identified as posing risk to a receptor. In the case of a local authority having multiple contaminated land sites, the contaminants have been expressed per site by listing the first few characters of the grid reference to specify which site is in question.
- *Document Links:* this is where any extra links to sources have been included, to make source accessibility easier for users of the dataset, these are commonly the remediation and determination statements that have been produced and published by the respective local authority.
- *Remediation Status:* this is the current status of the site, whether it has been remediated or if it is still classified as contaminated. Again, this has been (where necessary) made site-specific with the use of starting NGR characters. This is identified by the presence of a remediation verification statement, and in the case where no statement or suggestion of active works is present, it is assumed to be un-remediated.

A lot of columns have blank entries where there has been either no online register or where there are no determined sites on the register. The matrix can be filtered or sorted usefully, i.e. only local authorities with contaminated sites, only remediated sites or only sites with a certain contaminant present (hence being interactive). From the model, a nationwide picture of contaminated land can be produced. The total number of currently registered sites, site distribution, and the ratio of remediated to un-remediated sites are all examples of statistics that can be deduced from the data model. Note, again, that the deductions are based solely on publicly available data published by the local authorities responsible for that area. The information matrix (is shown in supplementary material Table 1) and the information on where what is present, and the current status of sites (in supplementary material Table 2).

From the perspective of seeking complete data amalgamation, the task is a challenging one, as relevant bodies up and down the country, while abiding by the same legislation, produce documents with clear differences in strategy, management and methods of presentation. The challenge is multi-layered, as it is not just the inequality in local authority datasets which affect the process of integration, but also the potential variation in the significance of a hazard. Currently, the information is basic, with the location of sites, contaminants and current status available to integrate. The sites in the matrix model can be further categorised into the types defined in the classification model developed.

### Conceptual GIS model

**Fig. 3:**
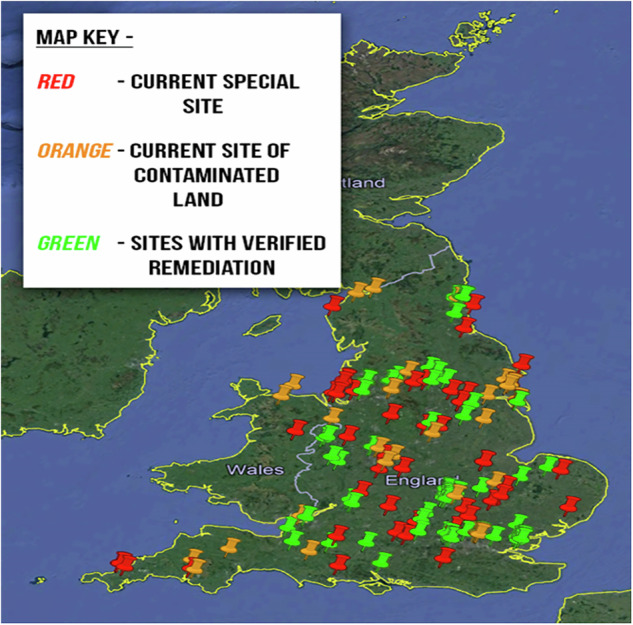
Full View of GIS Model of all Sites

**Fig. 4:**
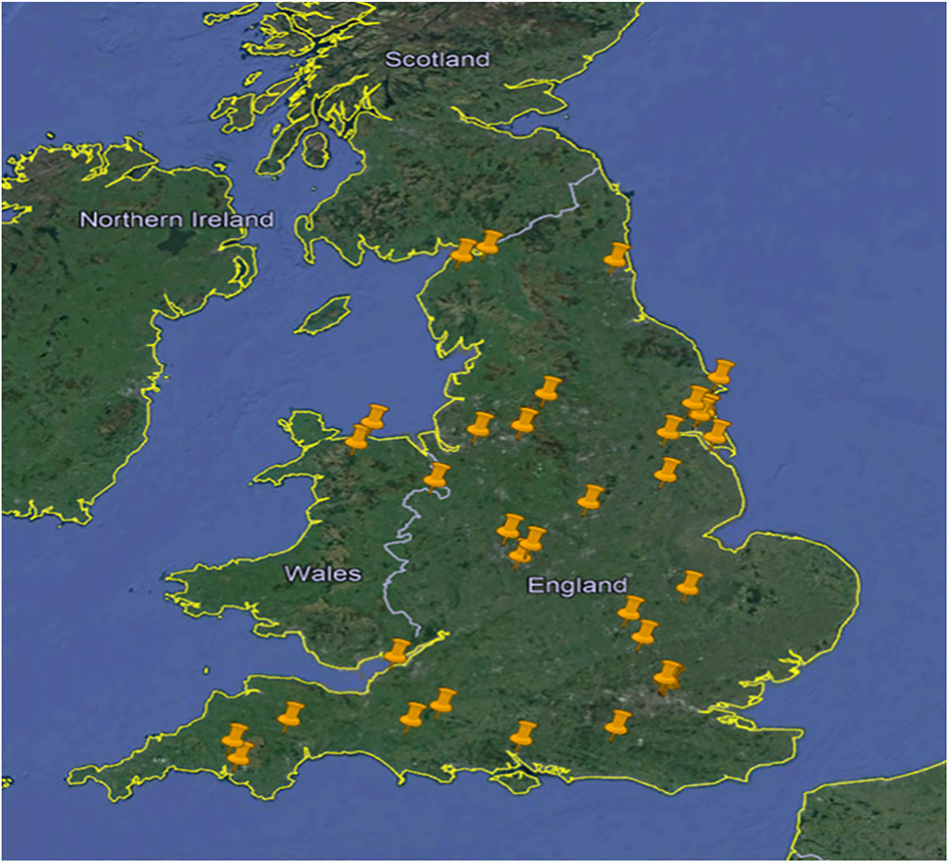
GIS Model of Only Current “Basic” Contaminated Sites

In this study, we have generated a conceptual model in GIS, applying the colour coding model shown in Fig. 2. Figure 3 shows a full view of GIS model of all sites, and Fig. 4 shows a GIS model of only current “Basic” contaminated sites. The categorised grid references (in supplementary material Table 3) allow for the input data into a national grid reference converter (Finder), this led to the creation of three GIS layers, one for each subset. These layers were overlaid onto the UK map, to give a visual description of each site included in online registers across England and Wales. It must be noted, some registers have grid references for every property, these are individually analysed and converted to appropriate localised grid references with an attached “number of properties” value. This removes bias within the GIS layer, as one register may describe a hundred houses with one grid reference, and another register: ten houses each with their own grid reference. Without localising the grid references to a generic site, the register with ten properties would appear more significant. This is an example of an issue posed by the non-standardised nature of documentation. The developed GIS layers have been refined repeatedly to a stage where they satisfactorily present the data available from contaminated land registers in England and Wales.

This innovative model provides an integrated overview of contaminated land in England and Wales. The model, like the underlying contaminated land database (the matrix), is interactive. The flexibility of zoom and movement on the model allows for focus onto individual sites, where potential receptor risks can be identified from the satellite imaging base map.

The GIS data can also be presented in different forms, one being the heat map shown in Fig. 1. This shows the spatial density of contaminated land sites - the concentration of contaminated land within England and Wales (concentration not of contaminants, but of sites per unit land area). The colour descriptive changes from transparent (at no sites) through to blue (low density), green (high density) and yellow at very high density. It can be seen from the heat map there are very high spatial concentrations in and around London, with other hotspots in the midlands.

**Fig. 5:**
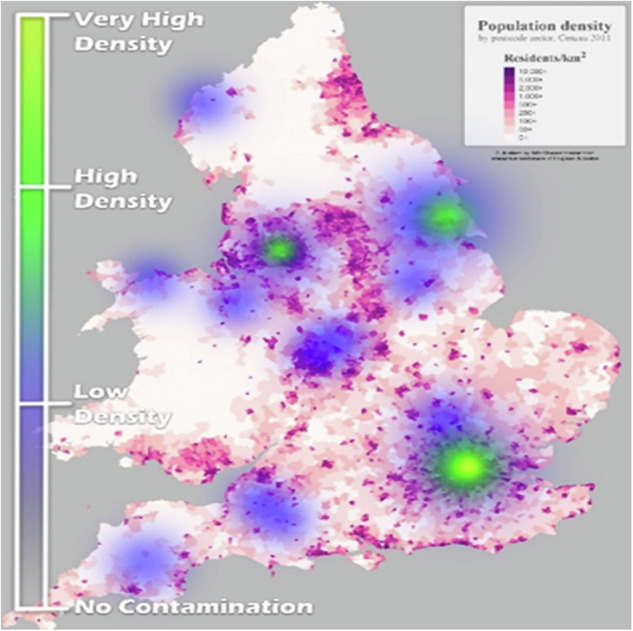
English and Welsh population density map plotted alongside the contaminated land density heat map from Fig. 1

While the heatmap (Fig. 1) clearly illustrates the spatial density of contaminated land sites across England and Wales, it is important to note that this visualisation is not a representation of the *total area* affected by contamination. As demonstrated by the numerical estimations in the Results and Discussion, the actual proportion of land impacted by formally determined contaminated land is relatively small, with approximately 0.0001% of land in England or Wales having once been, or still being, contaminated. The heatmap highlights areas with a higher *density* of contaminated sites, often correlating with historical industrial activity and urban centres (Fig. 5), but this does not equate to a widespread or continuous distribution of contamination. The majority of contaminated sites are in urban areas (70%), with 0.00027% of urban land estimated to be contaminated.

## Results and analysis

### Results from matrix model data

From the process of compiling the informational matrix in this study, it is apparent that different local authorities publish data about contaminated land (in their respective geographical areas) with significant disparities in practice. The potential for integration of public registers at a national level is limited, as local authorities have different approaches to collecting, documenting, and presenting data on potentially contaminated land and its remediation. The statistics and other findings interpreted from our model are collated in Table 1. Our model utilises a minimal set of data categories that are present in the majority of online contaminated land registers, thus allowing a national database to be developed, from which inferences can be made, as outlined in the following sections.

**Table 1:** Contaminated land statistics for online publicly available data for England and Wales

Category	Value	Unit	Notes/Description
Current Un-remediated Contaminated Land Sites (excluding Special Sites)	42	sites	Determined under Part 2 A of the Environmental Protection Act 1990.
Remediated Contaminated Land Sites (since 1990)	79	sites	On the public register.
Total Contaminated Land Sites Entered on Public Register (since 1990)	121	sites	Regardless of current status (excluding Special Sites).
Current Special Sites	53	sites	Designated as “Special Sites” due to serious risk.
Properties Affected by Contaminated Land (currently)	367	properties	Verified number of properties affected by contamination.
Properties Affected by Contaminated Land (since 1990)	1129	properties	Verified number of properties affected by contamination.
Councils/Boroughs with No Contaminated Land Sites on Public Register	118	councils/boroughs	Listed as having no entries on their public register.
Councils/Boroughs with No Data Available (Public Register Not Published)	178	councils/boroughs	No online record of the public register was found.
Councils/Boroughs with a Non-Zero Amount of Contaminated Land Sites	57	councils/boroughs	Council/borough with the most contaminated land sites on their public register
Council/Borough with the Most Contaminated Land Sites	13	sites	
Council/Borough with the Most Properties Affected by Contaminated Land	283	properties	
Councils/Boroughs with Benzo(a)pyrene Related Contamination	18	councils/boroughs	Out of the 57 councils/boroughs with contaminated land sites.
Councils/Boroughs with Arsenic-Related Contamination	10	councils/boroughs	Out of the 57 councils/boroughs with contaminated land sites.
Councils/Boroughs with Lead-Related Contamination	14	councils/boroughs	Out of the 57 councils/boroughs with contaminated land sites.
Council/Borough with the Most Special Sites	7	sites	

### Numerical estimations

As an example of the use of the matrix model to generate contaminated land statistics, we show how both areal extent of contamination and total exposed population can be generated at a national level using 'number of properties affected' (Table 1) as a proxy. It should be noted that this is illustative of the potential use of a national database and is based only on those public registers that were available online during the 2019 sampling period. The areal extent of contamination can be numerically derived using an assumed average property area. Furthermore, the number of people exposed to contaminated land could be estimated using an assumed number of residents per property. The average floor plan size of a detached house in the UK is around 86 square metres (EHS, 2018). This should be multiplied by a safety factor of 1.5 to account for the likelihood that some properties are farmland or larger industrial buildings. Therefore, the average property size for the purpose of this study is to be set at 129 square metres. Using the fact that 367 properties are currently affected by contaminated land (Table 1) and that the total land area of England and Wales is 151,149 square kilometres (Commonwealth, 2019), it can be estimated that:

- **47,343 square metres (0.00003%) of land in England and Wales is currently contaminated (2019 data)**.

Further, based on data since the introduction of Part 2 A of the Environmental Protection Act in 1990 (Table 1), in essence, all sites ever determined, then the statistics derived would be:

- **145,641 square metres (0.00001%) of land in England and Wales have once has been, or is still, contaminated (2019 data)**.

An important implication from this is that one-third of all determined contaminated land still exists, meaning only two-thirds has been remediated (2019 data). For the population exposed to contaminated land: there is an average of 2.4 people per the UK household (Statistics, 2017), multiplied by the same safety factor of 1.5, giving an average of 3.6 people per property. For some properties listed in the data collected, the number of people per property may be much higher, depending on the land use. Using this average it can be stated that:

- **An estimated 1320 people are currently exposed to contaminated land (2019 data)**.
- **An estimated 4065 people have been or still are, exposed to contaminated land (2019 data)**.

In calculating the number of people exposed, the uncertainties are high. This is because, unlike the spatial size of a property, there is such a high diversity of population densities when considering exposure. There are both rural and urban population densities, but it is the property type that drives the disparity in population densities. For example, the population density in a factory is high, whereas in a housing estate, it is relatively low. If the national average population densities are considered in parallel to the values estimated for total contaminated land area, it would only amount to around 20 persons exposed to contaminated land. It is important to note these values are likely to be underestimates due to the varying availability of information about some potentially contaminated sites on the public register. The are several factors which may result in an underestimation of the number of persons affected by contaminated land. Examples include: the lack of information availability in, or online accessibility to the public registers of more than half of local authorities in England and Wales; the underestimation of population densities in some cases; and the fact that exposure is not just affecting those who live there but also those who work there, walk by, or are nearby. These are the contaminated land sites which are not yet remediated, but which may pose a risk to humans, the environment and the economy.

### Inference from GIS model

The contaminated land GIS model shows the greatest presence of contaminated land in populated, ex-industrial areas e.g., London, Manchester, and Hull. This has been confirmed by overlaying the heat map presented in Fig. 1 onto a UK population density map as shown in Fig. 5.

The background choropleth map (Briskat, 2016) describes population density (in this case residents per square kilometre), where white is between 0 and 50 residents per square kilometre, and dark purple is over 10,000 residents per square kilometre. From the map, it can be seen around 70% of contaminated sites are in populated areas. The amount of contaminated land defined is significantly more important as the majority of it is within urban areas.

In the Table 2, values have been used to estimate the total amount of contaminated urban land, and the proportion of this to all urban land in England and Wales. 0.00027% of urban land is estimated to be contaminated. Due to their urban location, the receptor is likely to be humans.

**Table 2:** Area distribution of urban/built landmass, contaminated land, urban/built-up land contaminated

	Urban/built landmass out of England’s and Wales’s total landmass	Contaminated land sites in or near a built-up area	Urban/built-up land area that is contaminated
Percentage	8%	70%	0.00027%
Area	12,092 square km	0.033 square km	0.033 square km

Historically, Cornwall/Devon faced contamination from tin, copper, and arsenic mining, along with China Clay extraction, while North Wales suffered from slate and metal (lead, zinc, copper) mining. Other significant sectors contributing to contaminated sites across England and Wales include metal manufacturing, chemical industries, gasworks, landfills, and oil refineries (Government, 2014b). These sites are identified through preliminary assessments and historical land-use maps (GeoSmart, 2018b). Local authorities play a key role in investigating and managing these contaminated sites (Government, 2019). The model concept developed in this study can be used by both national and local government for statistic creation, future remediation planning, and visualising the current state of contaminated land in the UK to the general public. Providing similar maps of land-use history exist, comparisons can be made between the contaminated land model and the land-use models to better understand the historical influence on contaminated land. This could help identify patterns for site determination and in future identification of potentially contaminated land.

## Conclusion and recommendations

Contaminated land is a prevailing issue in England and Wales. This study has highlighted some limitations in the current legislative framework, and the subsequent challenges that are faced by a diverse range of associated stakeholders regarding contaminated land. The number of contaminated land designations are continually increasing. Although, at a comparatively slower rate, remediation efforts by local authorities, government agencies, environmental consultancies, and even responsible individuals are reducing this number. The study draws attention to the challenges faced in environmental communication related to contaminated land and proposes solutions at two levels - local and national.

Regarding management at a local authority level, inconsistencies have been identified concerning the organisation, presentation, and availability of contaminated land data. There is a wide disparity in the depth of information, the format, and even the online accessibility of such documents. The format of public registers is undefined in Part 2 A. Therefore, differences between local authorities in terms of the presentation and management of contaminated land records, cause complications in understanding, comparing, and integrating data. Moreover, modern decision making has a heavy dependency on information published online. In almost half of all local authorities, their public registers are not available online. To overcome these challenges, this study has developed and presented an innovative, conceptual data model to assist in streamlining the data management systems and practices of local authorities, thereby leading to the unification and standardisation of their public registers. This would improve the effectiveness of environmental communication and decision making.

On the other hand, at a national level, the suggested models can help devise and inform policies and strategies which could influence the redevelopment of contaminated land and the construction industry as a whole. The lack of integrated information in relation to contaminated land obstructs regulatory bodies such as the Environment Agency, and Natural Resources Wales, from working uniformly throughout the country. This study not only proposes the creation of a unified national dataset but also outlines three innovative, conceptual models, as follows:

1. An original classification model, which streamlines the categorisation of contaminated land.
2. A holistic matrix, which demonstrates the concept for the integration of information within local authority public registers into a single national entity.
3. A conceptual model that presents this integration of data in Fig. 2, as a GIS layer, displaying sites of current and previously contaminated land.

Such models, once fully developed, can complement local documents, leading to a holistic, national baseline for comparing similar contaminated land scenarios, between different parts of the country. This can also include other demographical and environmental GIS layers such as land-use, population density, and hydrology.

The findings from the paper can potentially be helpful for government bodies to review, redesign, and restructure contaminated land policy/strategy. Stakeholders in development and spatial planning will be able to benefit specifically from the GIS model, enabling them to more efficiently assess the contamination status of a given piece of land. Furthermore, these innovative models can be used effectively to inform the baseline study/preliminary investigation and the development of a Conceptual Site Model (CSM), which are crucial components of an environmental risk assessment. The models presented in this paper can be extended to an Environmental Impact Assessment (EIA) and Strategic Environmental Assessment (SEA), both at local and national levels, respectively. This study has the potential to contribute to the fulfilment of the Sustainable Development Goals (SDGs).

The usefulness of the GIS model could be enhanced by making site indicators interactive to present site-specific information. If within the models, risk levels (high, medium, and low) are assigned corresponding to the degree of contamination and the sensitivity of scenarios that could be an additional layer of information to assist decision making. Sites could also be classified by the current land-use of the site, such as industrial, commercial, or residential. Boundaries can be drawn around site indicators, to show the spatial extent of the site in question. The remit of both the matrix and GIS models could be expanded to include potentially contaminated sites in addition to the sites which are formally determined as contaminated land. In summary, the study paves the way for further research and development of these conceptual models to assist in the management of contaminated land.

## Supplementary information

Supplementary Materials

## Data Availability

There is no data outside this research paper to report. All the relevant data is included in the paper.
