# Supplementary material for: The Significance of Unifying Non-Integrated Information on Contaminated Land and Risks
Source: Environ Manage. 2025 Dec 27;76(2):51. doi: 10.1007/s00267-025-02334-8 (PMC12743671; doi:10.1007/s00267-025-02334-8)
Supplement: Supplementary file 1 — Supplementary Materials [file 267_2025_2334_MOESM1_ESM.docx]

**Table No. 1: Full View of CL Matrix Categories and Example Data**

| **Locations** | **Number of Basic Contaminated Sites** | **Number of Special Sites** | **Total Contaminated sites** | **Number of properties affected** | **Grid References** | **Contaminants** | **Document Links** | **Remediation Status** |
| --- | --- | --- | --- | --- | --- | --- | --- | --- |
| Castle Point Borough Council | [11](https://www.castlepoint.gov.uk/public-register-of-contaminated-land/) | 0 | 11 | 83 | TQ8117786871 (73 properties) TQ8009489419 TQ7913088821 TQ8175386860 TQ8026987542  TQ8009591487 TQ8030390380 TQ7638188420 TQ7891483563  TQ7700188594 TQ7638188420 | TQ811: PAH, lead TQ800: PAH, asbestos, petroleum TQ791: Petroleum TQ817: Petroleum TQ802: petroleum and PAH TQ8009: na TQ803: na TQ763:na TQ789: na TQ770: na rest: na | N/A | Remediated |
| Gateshead Metropolitan Borough Council | [4](https://www.gateshead.gov.uk/article/3502/Contaminated-land) | 0 | 4 | 4 | NZ268633 NZ2010062080 NZ234600 NZ209586 | NZ268: PCDD, PCDF, arsenic NZ201: hydrocarbon NZ234: methane and carbon dioxide NZ209: hydrocarbon | NZ268: https://www.gateshead.gov.uk/article/4268/Saltmeadows-Riverside NZ201: https://www.gateshead.gov.uk/article/4269/Swalwell-Depot-site-Whickham-Bank NZ234: https://www.gateshead.gov.uk/article/4260/Beggars-Wood-recreation-area NZ209: https://www.gateshead.gov.uk/article/4267/Dewhurst-Terrace | NZ268: Remediated NZ201: Remediated NZ234: Un-remediated NZ209: Remediated |
| London Borough of Hackney | [9](https://www.hackney.gov.uk/the-contaminated-land-regime) | 0 | 9 | 18 | TQ341874 (4 properties) TQ336868 (1 property) TQ330860 (1 property) TQ324858 (3 properties) TQ325859 (allotments) TQ339846 (allotment) TQ319873 (1 property) TQ341860 (3 properties) TQ352840 (2 properties) | TQ341: Lead TQ336: Lead and arsenic TQ330: Lead TQ324: Lead and arsenic TQ325: Lead and arsenic rest: No Data | Register: https://www.hackney.gov.uk/media/8328/Contaminated-land-register/spreadsheet/contaminated-land-register.xlsx?m=636506589376830000  determinations: https://www.hackney.gov.uk/media/3373/determinations-of-land-as-contaminated-land/zip/det-cont-land1.zip?m=636459238369900000 | TQ341: Remediated Others: Un-remediated |
| Newcastle-upon-Tyne City Council | [6](https://www.newcastle.gov.uk/services/environment-and-waste/environmental-health-and-pollution/contaminated-land/contaminated) | 0 | 6 | 6 | NZ2626064600 NZ2760063700 NZ2860064170 NZ2864064020 NZ2905063120 NZ2863062990 | NZ262: lead NZ276: Lead, arsenic, cadmium, nickel, benzo-a-pyrene  NZ2860: Lead, arsenic, cadmium and PCDD/ PCDF  NZ2864: Lead, arsenic, cadmium and PCDD/ PCDF  NZ290: Coal derived hydrocarbons NZ286: Lead and arsenic | N/A | Remediated |
| Shropshire Council - Unitary | [4](https://www.shropshire.gov.uk/media/9639/summary-of-contaminated-land-register.pdf) | 0 | 4 | 13 | SJ3700202505 SJ3945606068 SO5968968621 SO5161974305 | No Data | N/A | Remediated |

**Table No. 2: Specific View of Grid References, Contaminants, Document Links, and Remediation Status with Example Data(Finder, 2011)**

| **Locations** | **Grid References** | **Contaminants** | **Document Links** | **Remediation Status** |
| --- | --- | --- | --- | --- |
| Bassetlaw District Council | SK777941 SK741713 | SK777: Benzo(a)pyrene SK741: Benzo(a)pyrene | SK777: https://www.bassetlaw.gov.uk/media/2624/marshlanedetermination.pdf SK747: https://www.bassetlaw.gov.uk/media/2622/lincolnroaddetermination.pdf | Remediated |
| Huntingdonshire District Council | TL3170971961 TL1895359172 | TL189: kerosene TL189: benzo(a)pyrene | N/A | TL317: Un-remediated TL189: Remediated |
| Rochdale Metropolitan Borough Council | SD8318210562 SD8488211326 | SD831: Gases SD8488: lead, benz(a)pyrene and dioxins | SD831: http://www.rochdale.gov.uk/pests-pollution-and-food/contaminated-land/Pages/Boo-Hole-landfill-site.aspx SD848: <http://www.rochdale.gov.uk/pests-pollution-and-food/contaminated-land/Pages/Peel-Lane-contaminated-land.aspx> | Remediated |
| Southampton City Council | SU4298012760 SU429126 | SU4298: arsenic, lead, benzo(a)pyrene, explosive PAH's SU429: lead | SU4298: http://www.southampton.gov.uk/Images/Radcliffe%20(N)%20Completed%20Remediation%20Strategy%20Report%202_tcm63-366330.pdf SU429: <http://www.southampton.gov.uk/Images/Radcliffe%20(S)%20Completed%20Remediation%20Strategy%20Report_tcm63-366332.pdf> | SU4298: Un-remediated SU429: Remediated |

**Table No. 3: Subset (Types) Grid References**

| **Site with Verified Remediation** | **Current Contaminated Land Site** | **Current Special Site** |
| --- | --- | --- |
| SK777941 | NY1626343619 | TF1200003350 |
| SK741713 | NY325855548461 | TF1245002950 |
| TQ8175386860 | SP1530091100 | TL4320052400 |
| TQ8026987542 | SP1002279083 | TM1930049200 |
| TQ8030390380 | SD9956029389 | TA2462008920 |
| TQ203850 | SP9552841380 | TF9681707854 |
| SP8807034010 | SJ376635358813 | TL4481767637 |
| SP8760036730 | SK9640871476 | TL3569841174 |
